# Anion Transport Across Human Gallbladder Organoids and Monolayers

**DOI:** 10.3389/fphys.2022.882525

**Published:** 2022-05-24

**Authors:** Keyan Zarei, Ian M. Thornell, David A. Stoltz

**Affiliations:** ^1^ Department of Internal Medicine, University of Iowa Roy J. and Lucille A. Carver College of Medicine, Iowa City, IA, United States; ^2^ Department of Biomedical Engineering, University of Iowa Roy J. and Lucille A. Carver College of Medicine, Iowa City, IA, United States; ^3^ Department of Molecular Physiology and Biophysics, University of Iowa Roy J. and Lucille A. Carver College of Medicine, Iowa City, IA, United States; ^4^ Pappajohn Biomedical Institute, University of Iowa Roy J. and Lucille A. Carver College of Medicine,, Iowa City, IA, United States

**Keywords:** Gallbladder, organoid, anion transport, cystic fibrosis, paracellular, electrophysiology

## Abstract

Fluid and anion secretion are important functions of the biliary tract. It has been established that cAMP regulates Na^+^ absorption through NHE3. However, mechanisms of gallbladder anion transport are less defined. We created organoids and organoid-derived monolayers from human gallbladder tissue to measure organoid swelling and transepithelial electrophysiology. In our *in vitro* models, forskolin-stimulation caused organoid swelling and increased transepithelial anion transport. Full organoid swelling required Cl^−^while changes in short-circuit current were HCO_3_^−^-dependent. Organoids and monolayers from an individual homozygous for the cystic fibrosis-causing *ΔF508 CFTR* mutation had no apical expression of CFTR and minimal changes in transepithelial current and conductance with forskolin treatment. However, organoid swelling remained intact. Dilution potential studies revealed that forskolin treatment increased the paracellular permeability to anions relative to cations. These data suggest a novel paracellular contribution to forskolin-stimulated fluid transport across the gallbladder epithelium.

## 1 Introduction

Fluid and anion secretion across epithelial cells are important functions of the biliary tract. Alkaline biliary secretions make up nearly a third of bile by volume (Tabibian et al., 2013; Housset et al., 2016; Boron and Boulpaep, 2017) and are thought to protect the underlying epithelium from the cytotoxic effects of bile acids (Beuers et al., 2012; Hohenester et al., 2012). Further, defects in anion transport are associated with cholangiopathies (Prieto et al., 1993; Lindblad et al., 1999; Colombo et al., 2002; Assis and Debray, 2017).

Fluid transport across the gallbladder epithelium is controlled by receptor-mediated second messenger cascades. Elevated cAMP, which occurs in response to postprandial secretin release, decreases Na^+^ absorption across epithelia thereby increasing fluid secretion (Donowitz and Welsh, 1986; Yun et al., 1997; Zizak et al., 1999; Mennone et al., 2001; Tabibian et al., 2013; Housset et al., 2016). To maintain electroneutrality, anion transport must also be affected by cAMP and studies suggest roles for both HCO_3_^−^ and Cl^−^ (Tabibian et al., 2013; Housset et al., 2016). The current model of anion secretion suggests that Cl^−^is secreted across the biliary epithelium through the cystic fibrosis transmembrane conductance regulator, CFTR, anion channel which is enriched in gallbladder compared to other organs (Strong et al., 1994; Alpini et al., 1997; Chinet et al., 1999; Dutta et al., 2011; Uhlen et al., 2015). Additionally, cAMP mediates cholangiocyte alkalinization of bile (Roberts et al., 1993) and it is thought that secreted Cl^−^is required for HCO_3_^−^ secretion by Cl^−^/HCO_3_^−^exchangers (Banales et al., 2006). Despite these important studies in the field, the exact mechanism of biliary anion secretion remains unclear.

Studies of biliary physiology have been limited by lack of representative *in vitro* models. Recently, the development of organoid technology has provided a breakthrough in studying the biliary system (Barker et al., 2007; Sato et al., 2009; Sato et al., 2011; Huch et al., 2013; Huch et al., 2015; Schutgens and Clevers, 2020). In this study, we set out to better understand the mechanism(s) of cAMP-mediated anion secretion across the human gallbladder epithelium using human organoids and organoid-derived monolayers. We hypothesized that cAMP-mediated stimulation by forskolin would cause human gallbladder organoids to swell.

## 2 Materials and Methods

*Human gallbladder tissue acquisition.* Human gallbladder tissue was obtained through the University of Iowa Tissue Procurement core from donors undergoing elective cholecystectomies. In total, seven different human gallbladder specimens were obtained from otherwise healthy individuals undergoing elective cholecystectomies. One gallbladder specimen was obtained from an individual homozygous for the *ΔF508* mutation. Gallbladder samples were placed in cold William’s E media supplemented with nicotinamide (10 mM, Sigma), sodium bicarbonate (17 mM, Sigma), 2-phospho-l-ascorbic acid trisodium (0.2 mM, Sigma), sodium pyruvate (6.3 mM, Sigma), glucose (14 mM, Sigma), HEPES (20 mM, Sigma), dexamethasone (100 nM), insulin-transferrin-selenous acid (ITS) premix (1:100, Corning), penicillin-streptomycin (100 U/mL; 100 ug/mL, Gibco). This media will henceforth be referred to as supplemented William’s E media. Tissues were kept at 4°C for less than 24 h until the sample could be processed.

*Development of human gallbladder organoid model.* Methodology for the organoid model was adapted from a published protocol for human gallbladder tissue (Sampaziotis et al., 2017b). The luminal surface of the gallbladder tissue was scraped using sterile scalpels in William’s E media. Cells were centrifuged down at 440 *g* for 5 min. The cell pellet was washed with supplemented William’s E media and centrifuged down again at 440 *g* for 5 min. The resulting cell pellet was resuspended in supplemented William’s E media containing the following growth factors: 500 ng/ml human recombinant R-spondin 1 (R&D) and 40 ng/ml human epidermal growth factor (R&D). This growth factor-containing media is referred to as gallbladder organoid media. For the initial plating of the cells, 10 μM Y-27632 dihydrochloride (Tocris) was also added to the media but was not included for subsequent media changes. The cell pellet was mechanically dissociated so that there were cell clusters of approximately 5–20 cells. Two parts by volume Matrigel (Corning) was added to the cell suspension and the final mixture was plated in prewarmed 6-well or 24-well culture plates in small, 10–20 µL drops. The plates were inverted and incubated for 10–20 min at room temperature and then for another 30–40 min at 37°C. 400 μL (24-well) or 1 ml (6-well) of prewarmed gallbladder organoid media was then added to each well. Media was changed every 3–4 days. Organoids were passaged approximately once every 2–3 weeks: organoids were mechanically dissociated from Matrigel using cold William’s E media, centrifuged at 440 *g* for 5 min, and re-plated at a dilution of 1:5.

*Organoid swelling assay.* All experiments were done on the Zeiss LSM-880 multiphoton with temperature control (37°C) and humidity chamber. Gallbladder organoids were plated on glass-bottom culture dishes (Lab-Tek) 3–4 days prior to imaging. Approximately 30 min prior to the experiment, media was aspirated and replaced with Krebs-Ringer solution (118.9 mM NaCl, 25 mM NaHCO_3_, 1.2 mM CaCl_2_, 1.2 mM MgCl_2_, 2.4 mm K_2_HPO_4_, 0.6 mm KH_2_PO_4_, 5 mM dextrose in 5% CO_2_ (vol/vol), pH = 7.4), Cl^−^-free ringer solution (118.9 mM sodium gluconate, 25 mM NaHCO_3_, 2.4 mm K_2_HPO_4_, 0.6 mm KH_2_PO_4_, 5 mM calcium gluconate, 1 mM magnesium gluconate, 5 mM dextrose in 5% CO_2_ (vol/vol), pH = 7.4), HCO_3_^−^-free ringer solution (135 mM NaCl, 1.2 mM CaCl_2_, 1.2 mM MgCl_2_, 2.4 mm K_2_HPO_4_, 0.6 mm KH_2_PO_4_, 5 mM HEPES, 5 mM dextrose, pH = 7.4), or Cl^−^-free and HCO_3_^−^-free solution (143.9 mM sodium gluconate, 2.4 mm K_2_HPO_4_, 0.6 mm KH_2_PO_4_, 5 mM calcium gluconate, 1 mM magnesium gluconate, 5 mM dextrose, pH = 7.4). Baseline measurements of 10 organoids per condition were obtained. Forskolin (10 μM) or DMSO was added to the Ringer’s solution and organoids were monitored for 1 h with an image acquisition interval of 10 min. Whole organoid areas were obtained by hand tracing the images in Fiji. Organoid measurements were normalized to organoids in Krebs’/HCO_3_^−^ solution with DMSO. After this initial validation of our methodology, we subsequently used calcein green to conduct our studies more efficiently. We followed a protocol established by others (Boj et al., 2017). Briefly, on the day of the experiment, one vial of calcein green (50 μg; Invitrogen, Thermo Fischer) was thawed and dissolved in 5.1 μL of DMSO. 2.5 μL of the resuspended calcein green was added to 580 μL of organoid media. 10 μL of this final solution was then added to each well for imaging and allowed to incubate for 30 min. The imaging protocol described above was followed; after image acquisition, segmentation and area measurement for each well was done through the Zen software (Zeiss, RRID: SCR_013672) and reported as a total area per well. For CF organoids, we did not observe a difference in swelling for the different organoid morphologies and measured total area.

*Organoid immunocytochemistry.* Organoid immunocytochemistry was based on a previous study (Dekkers et al., 2019). Gallbladder organoids were mechanically dissociated from the Matrigel, washed, and fixed in 4% PFA for 15 min at 4°C. Organoids were kept in suspension at 4°C while they were permeabilized in 0.3% Triton for 1 h, and blocked in Superblock (Thermo-Fisher) with 4% normal goat serum for 48 h. Organoids were then incubated with primary antibodies overnight: mouse anti-CFTR (clone 769) (1:100 dilution, University of North Carolina—Chapel Hill and the Cystic Fibrosis Foundation Therapeutics, Cat# UNC769, RRID: AB_2904617) and rabbit anti-Na^+^/K^+^-ATPase (clone EP 1845Y) (1:100 dilution, Abcam, Cat# ab76020, RRID: AB_1310695). Organoids were then washed with PBS and incubated overnight with secondary antibodies goat anti-mouse conjugated to Alexa-Fluor 488 and goat anti-rabbit Alexa-Fluor 568 (Thermo Fisher Scientific, Cat# A-11001, RRID:AB_2534069). Organoids were then washed once again and incubated with Alexa-Fluor 633 conjugated phalloidin (Thermo Fisher Scientific, Cat# A22284) for 1 h at room temperature. Organoids were then washed in PBS, mounted with Vectashield plus DAPI (Vector Labs), coverslipped, and visualized with an Olympus Fluoview FV1000 confocal microscope 60x oil lens.

*Human gallbladder organoid-derived monolayers.* Gallbladder organoids were mechanically dissociated from Matrigel and spun down at 440 g at 4°C. The cell pellet was then resuspended in TrypLE (Gibco), incubated for 10 s in a 37°C water bath, and mechanically dissociated by pipetting up and down 10 times. The suspension was then viewed under a microscope and the procedure was repeated until a near single-cell suspension was achieved. At this point, William’s E media supplemented with 2% fetal bovine serum was added to neutralize the TrypLE, cells were spun down at 440 g at 4°C, washed with William’s E media, and spun down again. The cell pellet was then resuspended in gallbladder organoid media containing 10 μM Y-27632 dihydrochloride and 10 μM CHIR99021(Tocris) at a density of approximately 2.5 × 10^5^ cells/100 µL. Transwell permeabilized supports (Costar) were coated with a 1:20 dilution of Matrigel for at least 1 h at 37°C and washed with PBS prior to the plating of cells. 100 µL of cell suspension was added to each well for a seeding density of 2.5 × 10^5^ cells per well.

*Organoid-derived monolayer immunocytochemistry.* Monolayers were fixed in 4% PFA for 15 min at 4°C, washed with PBS for 1 h, permeabilized with 0.3% Triton for 15 min and then blocked in Superblock (Thermo-Fisher) with 4% normal goat serum overnight. Organoids were then incubated with primary antibodies overnight at 4°C: mouse anti-CFTR (clone 769) (1:100 dilution, University of North Carolina—Chapel Hill and the Cystic Fibrosis Foundation Therapeutics, Cat# UNC769, RRID: AB_2904617), and/or rabbit anti-Na^+^/K^+^-ATPase (clone EP 1845Y) (1:100 dilution, Abcam, Cat# ab76020, RRID: AB_1310695), and/or rabbit anti-NHE3 (1:100 dilution, Abcam, Cat# ab95299, RRID: AB_10674068). Organoids were then washed with PBS and incubated with goat anti-mouse and goat anti-rabbit secondary antibodies conjugated to Alexa-Fluor 488 and Alexa-Fluor 568 (Thermo Fisher Scientific, Cat# A-11001, RRID:AB_2534069) respectively for 1 h at room temperature. Organoids were then washed in PBS, mounted with Vectashield plus DAPI (Vector Labs), coverslipped, and visualized with an Olympus Fluoview FV1000 confocal microscope 60x oil lens.

*Ussing chamber studies.* For electrophysiology studies, monolayer cultures were mounted in Ussing chambers (Physiologic Instruments) and bathed in Krebs-Ringer solution (118.9 mM NaCl, 25 mM NaHCO_3_, 1.2 mM CaCl_2_, 1.2 mM MgCl_2_, 2.4 mm K_2_HPO_4_, 0.6 mm KH_2_PO_4_, 5 mM Dextrose in 5% CO_2_ (vol/vol), pH = 7.4), Cl^−^-free ringer solution (118.9 mM sodium gluconate, 25 mM NaHCO_3_, 2.4 mm K_2_HPO_4_, 0.6 mm KH_2_PO_4_, 5 mM calcium gluconate, 1 mM magnesium gluconate, 5 mM dextrose in 5% CO_2_ (vol/vol), pH = 7.4), HCO_3_^−^-free ringer solution (135 mM NaCl, 1.2 mM CaCl_2_, 1.2 mM MgCl_2_, 2.4 mm K_2_HPO_4_, 0.6 mm KH_2_PO_4_, 5 mM HEPES, 5 mM dextrose, pH = 7.4), or Cl^−^-free and HCO_3_^−^-free solution (143.9 mM sodium gluconate, 2.4 mm K_2_HPO_4_, 0.6 mm KH_2_PO_4_, 5 mM calcium gluconate, 1 mM magnesium gluconate, 5 mM dextrose, pH = 7.4) on both the apical and basolateral sides, as previously described (Chen et al., 2010). The final concentrations of the chemicals were the following: forskolin (apical—10 μM), GlyH (apical—100 μM), and DIDS (apical—100 μM).

*Dilution potential studies*. Dilution potential studies were calculated as described previously (Thornell et al., 2020). Briefly, dilution potential solutions were created with the following composition: 5 mM glucose, 1.2 mM calcium gluconate, 1.2 mM magnesium gluconate, 5 mM HEPES (pH 7.4). NaCl was made at the following concentrations in mM: 150, 112.5, 75, 37.5, 18.75, and were gassed with compressed air. All solutions were made to approximately 310 mOsm by mannitol addition and verified by vapor pressure (Wescor Inc.) for every experiment. Epithelia were assayed in Ussing chambers recording open-circuit transepithelial voltage where a 5 μA bipolar current pulse, applied periodically to the epithelium, was used to induce a change in transepithelial voltage (V_t_). The change in V_t_ was subsequently used to calculate the transepithelial conductance. Dilution potentials were generated by perfusing dilutions of the NaCl-containing dilution potential solutions above into the apical chamber. Electrode drift was assessed by lysing the cells with distilled water at the end of the experiment; afterwards, junction potentials from the ionic dilutions were assessed and subtracted from the obtained dilution potentials. The partial conductance, relative ion permeability (P_Cl_/P_Na_), and absolute ion permeabilities were calculated as describe previously (Thornell et al., 2020).

*Statistics.* All statistics were conducted using Graphpad Prism software (RRID:SCR_002,798). Non-parametric t-tests (Mann-Whitney), paired t-tests, or one-way ANOVA analyses were conducted when appropriate. *p* < 0.05 was considered statistically significant.

## 3 Results

### 3.1 Complete cAMP-Induced Swelling of Gallbladder Organoids Requires Cl^−^

**FIGURE 1:**
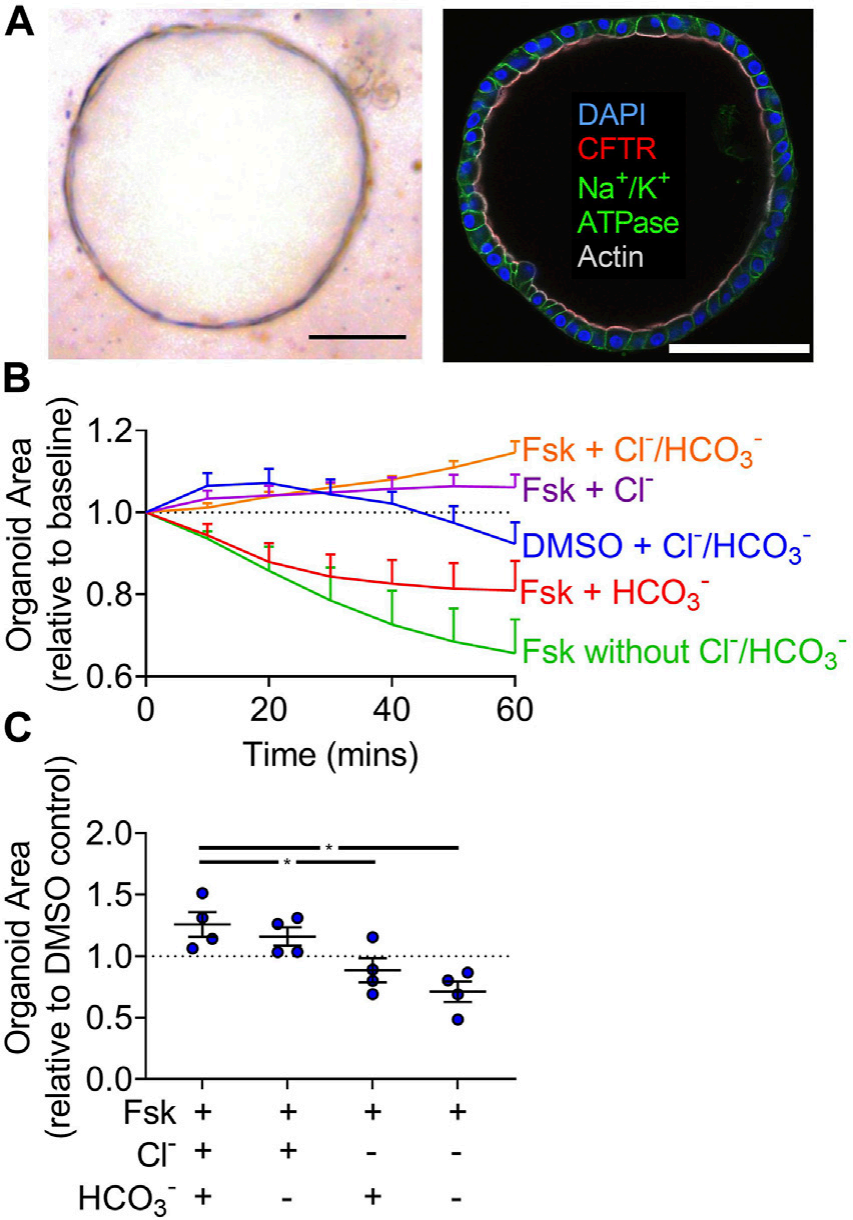
Full cAMP-induced swelling of gallbladder organoids requires Cl^−^. **(A)** Brightfield image and immunofluorescence (scale bar = 100 µm) of a human gallbladder epithelial organoid (scale bar = 100 µm). **(B)** Organoid area normalized to baseline over 1 h in Krebs’/HCO_3_^−^ + vehicle and Krebs’/HCO_3_^−^, HCO_3_^−^-free, Cl^−^-free, Cl^−^/HCO_3_^−^-free + 10 µM forskolin (Fsk). Each line represents the average of four donors. Bars represent SEM. **(C)** Individual donor values for 1 h time-point normalized to DMSO control for that donor in Krebs’/HCO_3_^−^. Bars indicate mean ± SEM. * indicates *p*-value<0.05; one-way ANOVA with Dunnett correction for multiple comparisons.

To study liquid transport across human gallbladder epithelia, we created organoids based on a study by Sampaziotis and colleagues (Sampaziotis et al., 2017a and 2017b). Human gallbladder organoids formed cystic structures and expressed CFTR along the lumen (Figure 1A). Relative to the DMSO control, organoids treated with forskolin increased in size by approximately 10% after 1 h (Figures 1B,C). The time-scale of organoid swelling in our study is similar to other studies in a variety of tissues (Sampaziotis et al., 2017a; Berkers et al., 2019; Sachs et al., 2019; Soroka et al., 2019). To assess the individual contribution of each anion, we performed gluconate substitution experiments. With complete gluconate substitution, organoids shrunk. This observation is consistent with forskolin-induced shrinking observed for CF organoids with impaired anion transport (Dekkers et al., 2013; Brewington et al., 2018; Xia et al., 2021). When compared to conditions containing both anions, Cl^−^-only containing solutions supported similar swelling but HCO_3_^−^-only containing solutions did not.

### 3.2 Gallbladder Organoid-Derived Monolayers Actively Transport Cl^−^ at Baseline

**FIGURE 2:**
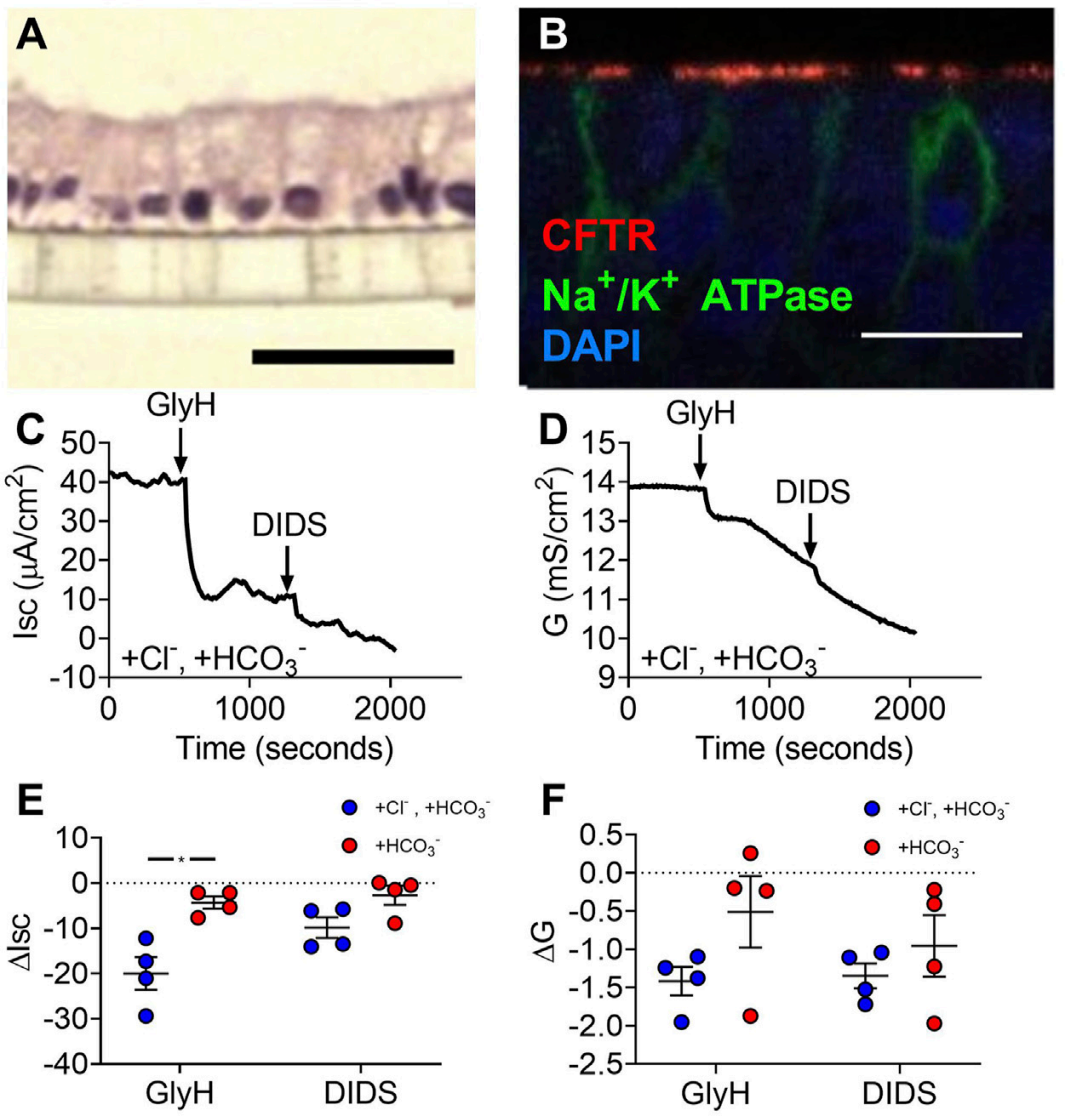
Gallbladder organoid-derived monolayers have unstimulated Cl^−^-transport at baseline. **(A)** H&E (scale bar = 40 µm) and **(B)** immunofluorescence (scale bar = 100 µm) of human gallbladder epithelial organoid-derived monolayer cultures. Representative **(C)** short-circuit current and **(D)** conductance traces and **(E)** ΔIsc and **(F)** ΔG in Krebs’/HCO_3_^−^ solution and Cl^−^-free solution. Bars indicate mean ± SEM. * indicates *p*-value<0.05; multiple t-tests with Holm-Sidak correction for multiple comparisons.

To assess active transport across gallbladder epithelia, we created organoid-derived monolayer cultures. These monolayers formed a simple columnar epithelium (Figure 2A) like gallbladder tissue and expressed apical CFTR (Figure 2B) and NHE3 (Supplemental Figure 1). Monolayers had short-circuit currents (Isc) and conductances (G) consistent with active transport across a leaky epithelia. Adding GlyH-101, a CFTR inhibitor, and DIDS, a non-selective inhibitor of other Cl^−^channels, to monolayer cultures decreased Isc and G **(** Figures 2C–F **)**. In Cl^−^-free conditions, GlyH-101 and DIDS decreased Isc and G but to a lesser degree than when Cl^−^was present (Figures 2C–F). These findings are consistent with epithelia that mainly perform active Cl^−^transport.

### 3.3 Gallbladder Organoid-Derived Monolayers Actively Transport HCO_3_^−^ When Stimulated With Forskolin

**FIGURE 3:**
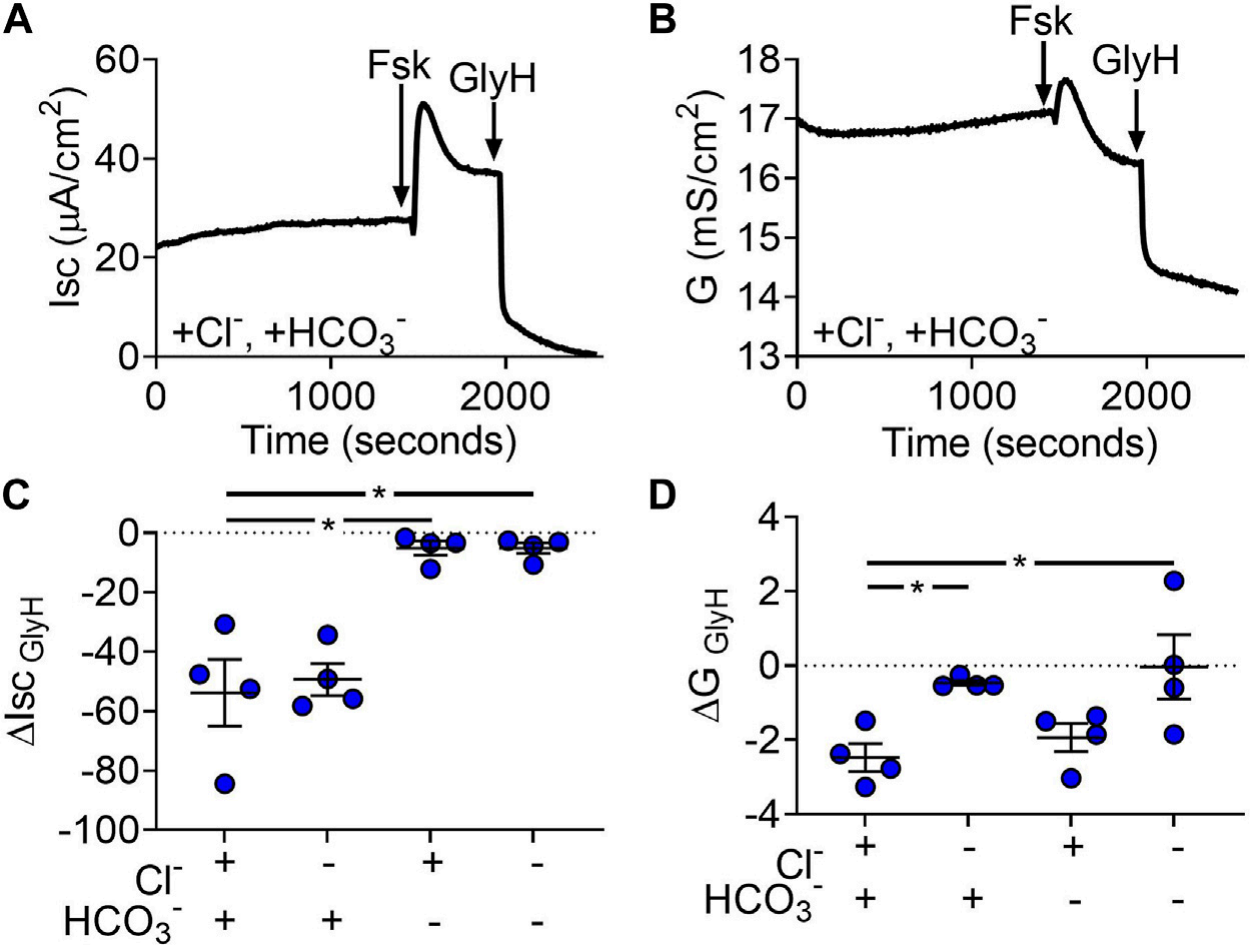
Gallbladder organoid-derived monolayers transport Cl^−^and HCO_3_^−^. Representative **(A)** short-circuit current (Isc) and **(B)** conductance (G) traces after apical addition of forskolin (final concentration = 10 µM) and GlyH (final concentration = 100 µM) **(C)** ΔIsc and **(D)** ΔG response to apical GlyH addition. Each symbol represents a different donor. Bars represent mean ± SEM. * indicates *p*-value<0.05; one-way ANOVA with Dunnett correction for multiple comparisons.

The gallbladder uses second messenger pathways that increase cAMP to secrete fluid (Chinet et al., 1999; Tabibian et al., 2013; Housset et al., 2016). Therefore, we next evaluated the ion-dependence of electrogenic transport after forskolin treatment. We hypothesized that Cl^−^secretion would be stimulated by forskolin based on the observation that forskolin caused gallbladder organoids to swell. In Cl^−^/HCO_3_^−^ solutions, forskolin caused a biphasic change in Isc and conductance (Figures 3A,B). To assess ion-dependences, we analyzed GlyH-101 inhibition after forskolin-induced changes were at steady-state. With forskolin present, the Isc was generated by HCO_3_^−^ as opposed to Cl^−^ (Figure 3C). Conductance analysis indicated that CFTR was still more permeable to Cl^−^rather than HCO_3_^−^ (Figure 3D). Contrary to our hypothesis, these data suggest that elevation of cAMP reduces the driving force for Cl^−^secretion and increases the driving force for HCO_3_^−^ secretion through CFTR.

### 3.4 Forskolin Treatment Increases Paracellular Permeability of Organoid-Derived Monolayers for Cl^−^Relative to Na^+^

**FIGURE 4:**
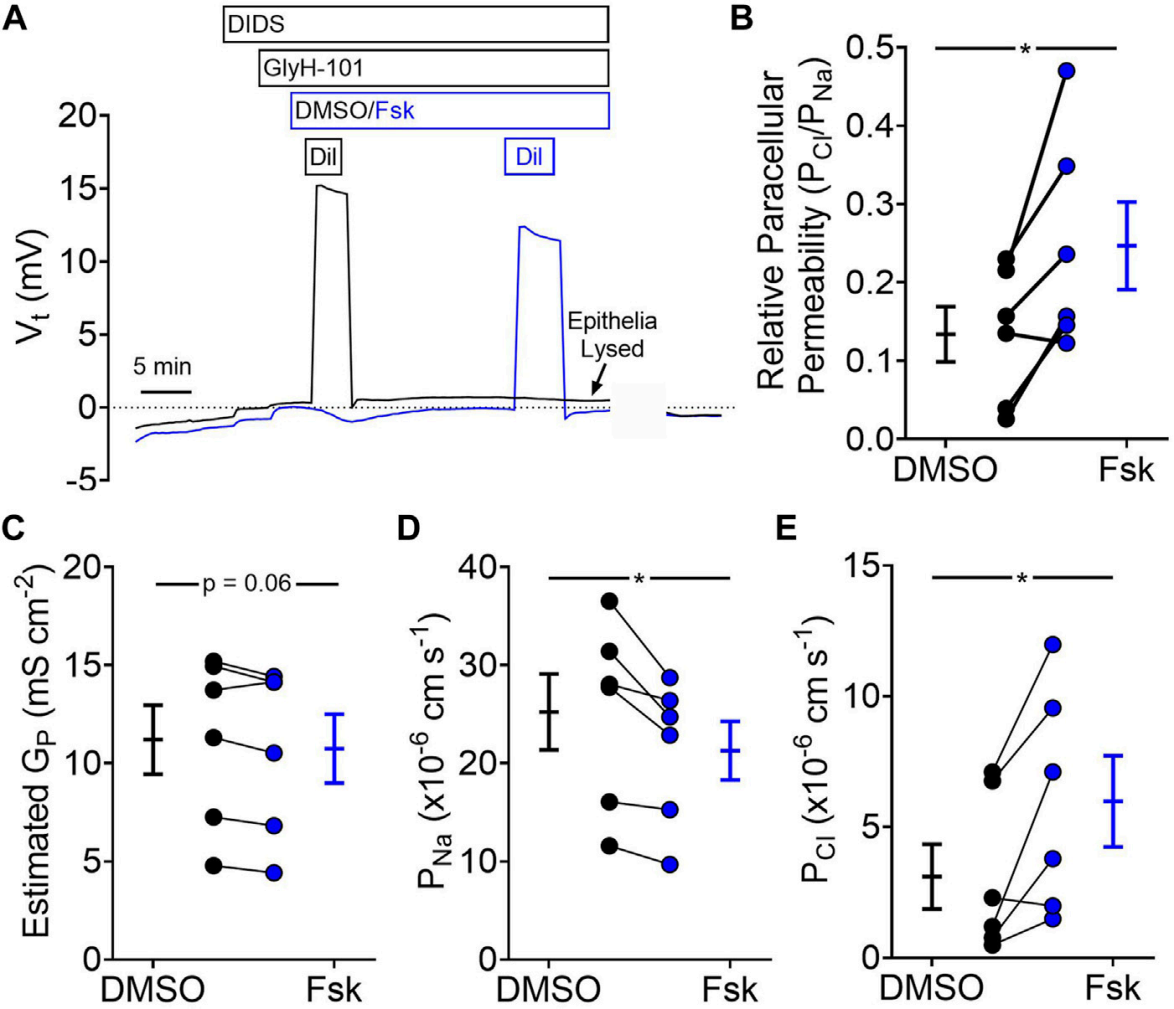
Forskolin increases paracellular permeability of Cl^−^relative to Na^+^ **(A)** Representative traces of voltage potential from dilution potential experiments. **(B)** Relative paracellular permeability of Cl^−^relative to Na^+^ after vehicle (DMSO) and forskolin (Fsk) treatment **(C)** Estimated paracellular conductance. **(D)** Na^+^ permeability, and **(E)** Cl^−^permeability before and after forskolin treatment. Final concentration: Fsk = 10 µM (apical), GlyH-101 = 100 μM, DIDS = 100 µM. Each symbol represents a different donor. Black = DMSO treated, Blue = Fsk. Lines connecting symbols indicating paired samples from the same donor. Bars indicate mean ± SEM. * indicates *p*-value<0.05, paired t-tests.

We found that the presence of Cl^−^was sufficient for forskolin-mediated organoid swelling. For short-circuited epithelia, forskolin elicited a large conductance change with a nominal change in short-circuit current. These data are consistent with the passive transcellular movement of Cl^−^. Passive ion movement is usually associate with the paracellular pathway, which lies in parallel with transcellular Cl^−^transport. To assess the role of paracellular transport, we measured potential differences generated by diluting apical NaCl across organoid-derived monolayers and calculated paracellular permeabilities for Na^+^ and Cl^−^ (Figure 4A). We found that, relative to vehicle, forskolin treatment increased the relative paracellular permeability of Cl^−^to Na^+^ (Figure 4B). On average, forskolin treatment did not alter the total paracellular conductance of the organoid-derived monolayers (Figure 4C); rather, Na^+^ permeability decreased (Figure 4D) while Cl^−^permeability increased (Figure 4E). These data suggest that elevated cAMP would decrease paracellular Na^+^ secretion and increase paracellular Cl^−^absorption across gallbladder with a lumen-negative voltage and in the absence of ion gradients.

### 3.5 Forskolin Treatment Induces Swelling in *ΔF508* Organoids but has Minimal Effects on *ΔF508* Organoid-Derived Monolayer Electrophysiology

**FIGURE 5:**
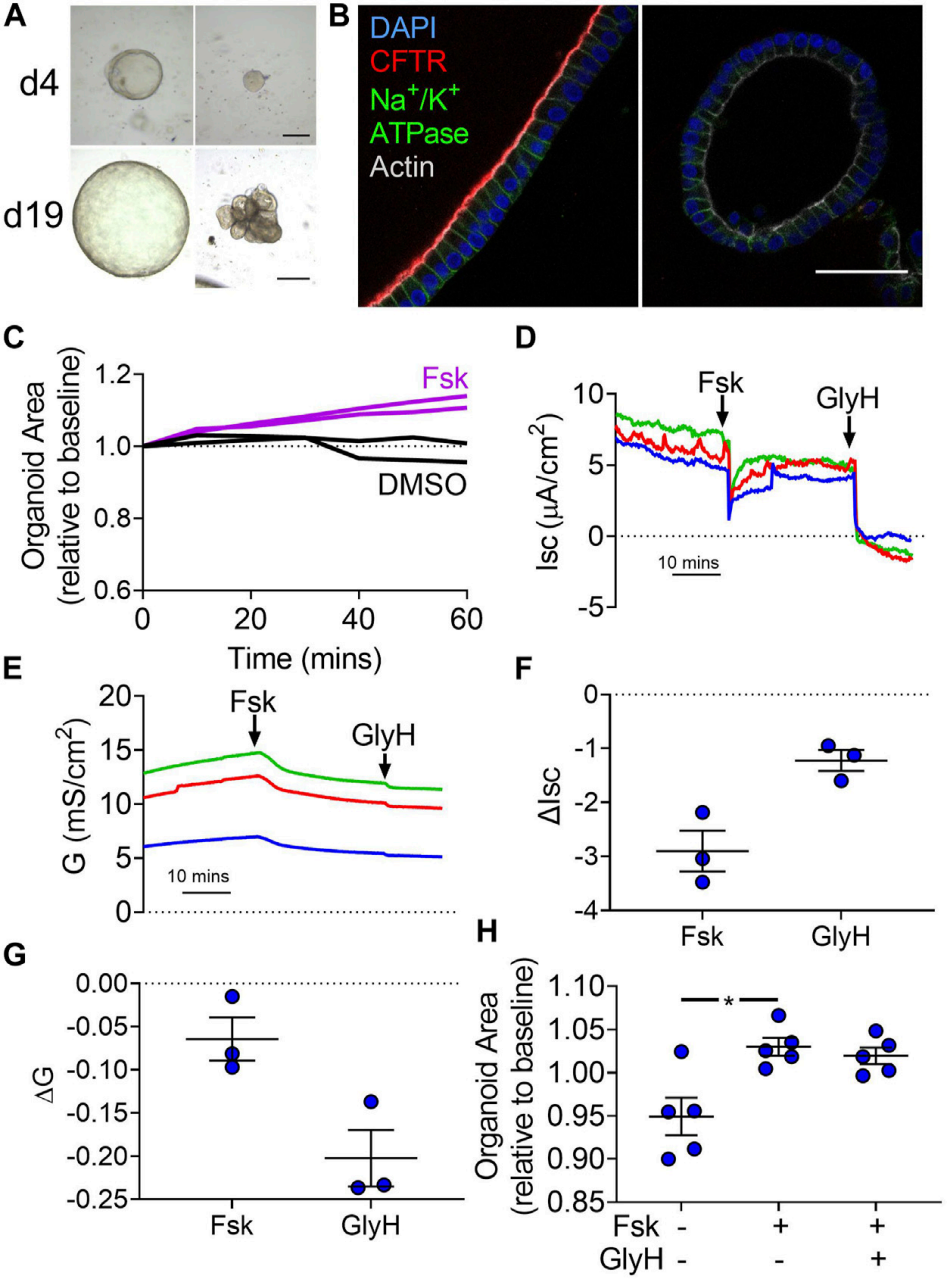
Forskolin induces swelling of *ΔF508/ΔF508* organoids without electrophysiological changes in organoid-derived monolayers. **(A)** Representative brightfield images of *ΔF508/ΔF508* organoids on days 4 (d4) and 19 (d19) of culture (passage 0). Some organoids demonstrated morphology like the non-CF donors (left panels) while other organoids demonstrated budding and lumen irregularities. **(B)** Apical CFTR immunofluorescence (red) was present in non-CF donors (left panel) while not present in the *ΔF508/ΔF508* organoids (right). Despite lack of CFTR expression, *ΔF508/ΔF508* demonstrated apical-basolateral polarity. Blue—DAPI. Green—Na^+^/K^+^-ATPase. Gray—Actin (scale bar = 50 µm). **(C)** CF organoid swelling with 10 µM forskolin (Fsk) or vehicle (DMSO) treatment. Data represent two experiments with different seedings of the CF donor. **(D)** Isc and **(E)** G traces of three different seedings (indicated by colors) in Krebs’/HCO_3_^−^ solution. **(F)** ΔIsc and **(G)** ΔG values for three different seedings (Final concentration: Fsk = forskolin 10 µM (apical), GlyH−101 = 100 µM) **(H)** Non-CF organoid forskolin-induced swelling after 1 h pretreatment with either DMSO or GlyH. Each symbol represents a unique donor. Bars represent mean ± SEM. * indicates *p*-value<0.05; one-way ANOVA with Dunnett correction for multiple comparisons.

Our data suggest that the cAMP-mediated increase in lumen volume requires Cl^−^. Our electrophysiology experiments with forskolin suggest that the transcellular pathway for Cl^−^becomes passive and the paracellular pathway increases its Cl^−^permeability. Therefore, we evaluated whether the paracellular pathway alone was sufficient for volume changes. To decrease the transcellular pathway for Cl^−^, we obtained gallbladder tissue from one person homozygous with the CF-causing *ΔF508 CFTR* allele. Cells from this donor formed organoids with a variety of morphologies. Some organoids, like those of other donors without *CFTR* mutations, had a single lumen surrounded by a layer of cells. Others had multiple budding structures without a clear central lumen (Figure 5A). Immunofluorescence of these organoids revealed no apical CFTR expression (Figure 5B). Forskolin induced a swelling response for organoids obtained from this CF donor (Figure 5C). Ussing studies revealed CF organoid-derived monolayers lacked forskolin-induced increases and GlyH-101-induced decreases in Isc and G, consistent with decreased CFTR activity (Figures 5D–G). As summarized in Supplementary Table S1, these data suggest that liquid transport across CF gallbladder epithelia remains intact despite nominal CFTR activity.

To further test the role of transcellular Cl^−^transport in organoid swelling, we pre-treated non-CF organoids with the CFTR inhibitor GlyH-101, which is membrane permeable (Sonawane et al., 2006). GlyH-101 did not affect the forskolin-stimulated swelling of organoids (Figure 5H). Taken together, these data suggest that CFTR plays an important role in electrophysiological changes induced by forskolin. However, Cl^−^permeability associated with the paracellular pathway is sufficient to support volume changes.

## 4 Discussion

In this study, we found that transcellular anion transport is controlled by cAMP. Isc across gallbladder epithelia was generated by Cl^−^secretion through CFTR. After applying forskolin, which increases cAMP, Isc was then generated by HCO_3_^−^ secretion through CFTR. The forskolin-mediated change in anion secretion did not reduce the CFTR conductance, thus cAMP altered the driving force for anions. Calu-3 cells also have greater CFTR-mediated HCO_3_^−^ vs Cl^−^short-circuit currents, which can be reversed by potentiating K^+^ channels to increase the driving force for Cl^−^secretion (Devor et al., 1999).

Complete forskolin-induced organoid swelling required Cl^−^and was unaffected by CFTR activity. A previously described role for AE2 in cAMP-mediated fluid secretion cannot explain our results because organoids bathed in the absence of HCO_3_^−^ still swelled. We speculate that Cl^−^passively secretes across the transcellular and paracellular pathway during forskolin stimulation. For the transcellular pathway, the large conductance change with minimal short-circuit current change suggests that the transcellular Cl^−^flux, which involves CFTR, is passive with forskolin. For the paracellular pathway, we observed an increase in the paracellular permeability to Cl^−^and decrease in paracellular permeability to Na^+^ after forskolin treatment. Changes in ion permeability were not accompanied by a conductance change, therefore forskolin altered the permselectivity of cultured gallbladder epithelia. Our data with a CF organoid and GlyH-101 treated organoids suggest that most of the passive Cl^−^flux is through the paracellular pathway. Consistent with a prominent role for paracellular transport in bile fluid transport, claudin-2 deficiency, a paracellular protein involved in paracellular transport, reduces bile flow in mice (Matsumoto et al., 2014).

What is the function of CFTR in the gallbladder? Pre-prandial, the gallbladder absorbs Na^+^ through NHE3 in the apical membrane and Na^+^/K^+^-ATPase in the basolateral membrane. Na^+^ absorption is balanced by transcellular and paracellular Cl^−^absorption and passive Na^+^ reflux through the paracellular pathway with the lumen-negative voltage provided by apical CFTR. Postprandial, CFTR may play two roles. First, CFTR may keep cellular Cl^−^in equilibrium with luminal Cl^−^, thus limiting the alkalinization achieved by AE2 (Tabibian et al., 2013; Housset et al., 2016) to match the cholangiocyte pH value, typically around 7.2 for cells. Accumulated intracellular Cl^−^would cause osmotic changes and regulatory volume decreases may occur over time. Second, cAMP-mediated HCO_3_^−^ secretion through CFTR in parallel to an increase in paracellular Cl^−^permeability would provide additional HCO_3_^−^ secretion to achieve pH values in excess of cholangiocyte pH values. Consistent with this notion, secreting gallbladder can reach luminal pH values greater than intracellular pH values (Martin et al., 1998; Chinet et al., 1999; Cuthbert, 2001).

Chinet et al. found that gallbladder cultured from humans have Ca^2+^-activated Cl^−^channels and that gallbladder cultured from people with CF lack forskolin-stimulated Isc, but have elevated Ca^2+^-activated Cl^−^channels (Chinet et al., 1999). Although we did not directly test Ca^2+^-activated Cl^−^channels, the forskolin response was not changed by GlyH, which also blocks some Ca^2+^-activated Cl^−^channels. This finding suggests that a GlyH-insensitive pathway that is not electrogenic exists for Cl^−^movement across gallbladder epithelia. Consistent with this idea, we found that forskolin increased the permeability of Cl^−^relative to Na^+^ through the paracellular pathway.

It may be relevant that although the gallbladder expresses high amounts of CFTR compared to other organs, only about 30% of people with CF present with a gallbladder disease (Assis and Debray, 2017). Our data suggests that CF gallbladder disease may be mild because of CFTR’s minor role in liquid transport and the presence of multiple HCO_3_^−^ secretion mechanisms. Consistent with this idea, several studies have provided evidence for contributions of both electroneutral and electrogenic HCO_3_^−^ secretion across gallbladder epithelia (Winterhager et al., 1986; Petersen et al., 1993; Martin et al., 1998; Moser et al., 2007; Tabibian et al., 2013; Housset et al., 2016). In addition, differential expression of disease modifier genes among people with CF may also play a role in variable CF gallbladder pathology (Bartlett et al., 2009; Debray et al., 2019).

Our previous studies determined the pig gallbladder organoids required either Cl^−^or HCO_3_^−^ for forskolin-induced swelling (Zarei et al., 2020). A species-related or age-related difference in cAMP-mediated anion transport may explain the contrasting results. Studies of human gallbladder tissue by Chinet et al. determined that only removal of both anions significantly decreased unstimulated or stimulated transepithelial current (Chinet et al., 1999). These differences may be in part due to fresh tissue versus cultured organoids. Future studies evaluating cultured epithelial responses to bile components and inflammatory mediators, such as prostaglandins, are warranted.

This study has several strengths: 1) we used novel *in vitro* models to explore gallbladder physiology; 2) we used several functional techniques to explore the physiological response to forskolin treatment; 3) we explored the individual role of Cl^−^and HCO_3_^−^ in gallbladder physiology; 4) we explored the forskolin-response of a *ΔF508/ΔF508* human gallbladder, a relatively rare tissue sample. Despite these advantages, our study also has several weaknesses: 1) we did not fully characterize transcript expression in our organoids and cultures, however organoids were formed using the protocol from Sampazoitis et al. (Sampazoitis et al., 2017); 2) we did not have genetic interventions to cleanly assess the function of various channels in gallbladder physiological response, specifically other anion channels; 3) we were unable to conduct studies with more than one CF gallbladder donor as it was difficult to obtain—thus, there is a possibility that our results represent an outlier; 4) forskolin is not a physiological stimulus—therefore, a stimulus like secretin, a gut hormone that also increases intracellular cAMP, may reveal variations in our results.

In these organoid and organoid-derived monolayers models, forskolin-stimulation led to swelling and increases in anion transport. We demonstrate that the human gallbladder transports both Cl^−^and HCO_3_^−^. We further suggest that forskolin-mediated organoid swelling, a proxy of fluid-secretion, may be in part due to paracellular anion and fluid transport.

## Data Availability

The raw data supporting the conclusions of this article will be made available by the authors, without undue reservation.
