# Supplementary material for: Anion Transport Across Human Gallbladder Organoids and Monolayers
Source: Front Physiol. 2022 May 24;13:882525. doi: 10.3389/fphys.2022.882525 (PMC9171199; doi:10.3389/fphys.2022.882525)
Supplement: Supplementary file 1 [file Table1.DOCX]

**Supplemental**

**Table 1: Summary of non-CF and CF organoid swelling and Ussing electrophysiology.** Organoid swelling [fold-change and standard deviation (SD)] from DMSO in Krebs'/Bicarbonate solution. Forskolin-(Fsk) and GlyH-101-induced (GlyH) ΔIsc (mean and SD) for non-CF and CF cultures in Krebs'/Bicarbonate solution. Swelling for the CF donor was within 1 standard deviation of the distribution for non-CF swelling.

|  | **non-CF (n=4)** | **CF (n = 1)** |
| --- | --- | --- |
| **Swelling** (fold-change ± SD) | 1.3 ± 1.1 | 0.20 |
| **GlyH-induced ΔIsc** (mean ± SD) | -53.8 ± 22.4 | -1.2 |
